# Impulsivity-Compulsivity Axis: Evidence of Its Clinical Validity to Individually Classify Subjects on the Use/Abuse of Information and Communication Technologies

**DOI:** 10.3389/fpsyg.2021.647682

**Published:** 2021-04-06

**Authors:** Daniel Cassú-Ponsatí, Eduardo J. Pedrero-Pérez, Sara Morales-Alonso, José María Ruiz-Sánchez de León

**Affiliations:** ^1^Data Analysis and Science, Operations Unit, Banco Santander, Madrid, Spain; ^2^Training and Research Unit, Evaluation and Quality Department, Madrid Salud, Madrid, Spain; ^3^Department of Experimental Psychology, Cognitive Processes and Speech Therapy, Faculty of Psychology, Complutense University of Madrid, Madrid, Spain

**Keywords:** addiction, ICT—information and communication technologies, impulsivity, compulsivity, machine learning

## Abstract

The compulsive habit model proposed by Everitt and Robbins has accumulated important empirical evidence. One of their proposals is the existence of an axis, on which each a person with a particular addiction can be located depending on the evolutionary moment of his/her addictive process. The objective of the present study is to contribute in addressing the identification of such axis, as few studies related to it have been published to date. To do so, the use/abuse of Information and Communication Technologies (ICT) was quantified on an initial sample of 807 subjects. Questionnaires were also delivered to measure impulsivity, compulsivity and symptoms of prefrontal dysfunction. Evidence of the existence of the proposed axis was obtained by means of Machine Learning techniques, thus allowing the classification of each subject along the continuum. The present study provides preliminary evidence of the existence of the Impulsivity-Compulsivity axis, as well as an IT tool so that each patient that starts getting treatment for an addiction can be statistically classified as “impulsive” or “compulsive.” This would allow the matching of each person with the most appropriate treatment depending on his/her moment in the addiction/abuse process, thus facilitating the individualized design of each therapeutic process and a possible improvement of the results of the treatment.

## Introduction

Based on the recent findings in Neuropsychology, various models have been proposed to rationalize addictive behavior (Ruiz-Sánchez de León and Pedrero-Pérez, 2019). Among them, one of the most empirically-supported one is the model of compulsive habit, which conceives addiction as a transition from the behavior controlled by the prefrontal cortex toward an habituated behavior executed by the ventral striatum and, finally, a compulsive habit governed by the dorsal striatum. This model, which was initially proposed by Everitt and Robbins (2005), was subsequently reformulated by the same authors (Everitt and Robbins, 2013, 2016) and has been recently updated to incorporate the findings reported by means of novel optogenetic stimulation methods (Lüscher et al., 2020). The model was formulated from the learning mechanisms that explain how some people that consume drugs proceed from a goal-orientated behavior (e.g., to obtain pleasure) to an habituated one in which the goal orientation no longer exists and reach, in some cases, the development of a compulsive habit that is maintained despite its dire consequences.

Previous studies exploring the role of impulsivity and compulsivity in addictive processes have given a key importance to impulsivity in the acquisition and maintenance of addiction, though they consider that compulsivity is a more recent and less studied concept (Yücel and Fontenelle, 2012). Impulsivity is generally defined as the tendency of acting in a fast and little-thought way, with no risk and consequences calculation (Rømer Thomsen et al., 2018) and, although there is still no agreement in considering the components of this concept, it is definitively multidimensional (Sperry et al., 2016). From a neurocognitive perspective, this concept is currently assumed to present, at least, three key elements: response inhibition, reward discounting and disadvantageous decision-making (Lee et al., 2019). Impulsivity is linked to a wide spectrum of disruptions, like pathological gambling, compulsive shopping, kleptomania, trichotillomania, skin picking, pyromania, social and borderline personality disorders, and binge eating, among others (Dell'Osso et al., 2006). The impulsive behavior is one of the central constructs in substance use disorders (SUD), with which not only conceptual aspects are shared, but also neurobiological substrates and sensibility to psychological and pharmacological treatments (Jupp and Dalley, 2014; Kozak et al., 2019).

Contrary, compulsivity is considered to be a relatively novel construct in the addiction context (Yücel and Fontenelle, 2012). Several authors have defined compulsivity as a multidimensional concept featured by: (a) poor response inhibition, which is also shared with impulsivity; (b) contingency-related cognitive inflexibility, (c) task or attentional set-shifting; (d) attentional bias or disengagement, that is, difficulty in disengaging from salient stimuli, and (e) habit learning, that is, the learning of a conditioned stimulus-response and, thus, insensitivity to the goals or consequences of actions (Lee et al., 2019). The complexity of this concept resides in the fact that it adds an affective component to urgency to execute behaviors oriented toward avoiding or reducing discomfort (Lee et al., 2019). The study of compulsivity is often limited to its relation with obsessive-compulsive disruption (Shephard et al., 2021), which can be considered the paradigmatic phenotypic manifestation, but not the only one, as it also seems to be a central element in food and pathological gambling disruptions, as well as in the so-called behavioral addictions (Fineberg et al., 2018). The treatment of problems associated to compulsivity include psychological interventions (more concretely, the exposure with response prevention), pharmacological therapies (selective serotonin reuptake inhibitors), and transcranial magnetic stimulation and neurofeedback, among others (Shephard et al., 2021).

While there is a growing interest in research to emphasize the differences between impulsive and compulsive behaviors, it is uncommon in clinical practice to consider such differences and the need of addressing them in a distinct way. Pharmacological treatments are formulated in accordance to the consumed drug and the available clinical evidence for each drug group (Portelli et al., 2020), while psychological treatments tend to be applied depending on the available diagnosed categories (Cabaniss and Holoshitz, 2019), but without considering the differential evolutionary moment for each person. The utility of this categorical approach has been vastly disputed in the recent years, thus resulting in a dimensional consideration of mental health problems (Gillan et al., 2016; Fineberg et al., 2018). In this sense, the Research Domain Criteria Project (RDoC; Insel et al., 2010) was devised in order to classify psychopathology in a new way to provide a better support for the development of treatments based on observable behavior dimensions with established biological validity, independently on the symptomatic diagnose in which the current categorical classifications are founded.

From this new point of view, addiction can be regarded as a process that affects the superior mechanisms of behavior, which are governed by different cerebral structures according to the individual evolutionary moment. The change of the cerebral location of the control of such behavior occurs gradually, similarly to a transition reinforced by repetition. In this way, each person that uses substances is located at some point along the continuum between the behavior controlled by the prefrontal cortex and the automated and compulsive behavior, executed from the dorsal striatum (van den Heuvel et al., 2010). The understanding of where each individual is located could help in providing the most appropriate treatments (psychological and pharmacological) in its evolutionary moment. Therefore, it would be of great interest to translate the findings of the model to the clinical area. There have been some previous attempts to establish the existence of a certain Impulsivity-Compulsivity axis, which have made it possible to find some indications of its existence based on the use of questionnaires and/or neuropsychological performance tests, both related to substance addictions (Fernández-Serrano et al., 2012).

The scope of the so-called behavioral addictions has experienced an explosive increase in the last two decades, especially owing to the widening of private Internet use and, subsequently, the emergence of smartphones and their associated apps. This fact can be stated by carrying out a rapid search on PubMed database with the words “behavioral addiction” as search criteria, which yields 601 works published in 1995, 944 in 2000 and 5,992 in 2019. Such behaviors, which are linked to the use of the Internet, have been grouped under the Information and Communication Technologies (ICT) term, though behavioral addictions involve many other behaviors, like pathological gambling, addition to food and many others that do not entail the use of substances (Grant et al., 2010). There is some controversy on the suitability of considering the addiction to such behaviors: while the number of studies applying the comprehensive frameworks of addiction to substances in this new behavior group keeps rising (i.e., Horvath et al., 2020), others seriously dispute the appropriateness of equating both problems from the biochemical level to the social consideration level (Billieux et al., 2015; Panova and Carbonell, 2018). However, it seems to be accepted that both behaviors share a location in the new spectrum of impulsive-compulsive disorders (Robbins and Clark, 2015).

Previous works, in which Machine Learning techniques were applied, proved the importance of the impulsivity and compulsivity dimensions in behavioral addictions, though no method was proposed to estimate the location of individuals at a certain time, aside from psychiatric qualifying systems (Ioannidis et al., 2016). From a similar theoretical perspective, Machine Learning techniques were also employed to estimate the gravity of the problematic use of smartphones or of any of their components (Elhai et al., 2020). The use of Machine Learning techniques in the study of aspects related to addictive behaviors (with or without substances) has progressively increased in the last years, thus yielding a valuable work that will be useful in future clinical applications (Mak et al., 2019). The use of such techniques has recently proliferated to study concrete aspects on the use/abuse of ICT (Di et al., 2019; Kamaruddin et al., 2019; Xu et al., 2019; Gross et al., 2020).

In general terms, the encountered works tend to focus on the symptoms used in diagnostic categories from a categorical perspective. RDoC Project proposes transitioning from systems based on symptomatic categories to dimensional neurocognitive models, which can be applied in the clinical area.

The aim of the present work was to provide new empirical evidence of the existence of an Impulsivity-Compulsivity axis based on the neurocognitive model proposed by Everitt and Robbins (2016), which would allow an efficient classification of each person with addictive behaviors at some point of the above-mentioned hypothetical continuum. To do so, descriptive and inferential statistical analyses, including Machine Learning techniques, were conducted on the outcome of questionnaires that estimate impulsivity, compulsivity, prefrontal functioning and use/abuse of ICT. Furthermore, based on the current clinical methodologies exposed above, we were encouraged to release an IT tool capable of providing a rapid assessment on the impulsive/compulsive character of individuals, thus allowing a more efficient diagnosis, a rapid clinical application, as well as an effective treatment assignation.

## Methodology

### Participants

A total sample of *n* = 812 subjects was obtained. After performing an outliers analysis, 5 subjects were excluded (0.6%), resulting in a final sample of *n* = 807 subjects.

A preliminary analysis to evaluate the demographics of the study population (Table 1) revealed that the sample was predominantly composed by women (67.7%) vs. men (32.3%), mainly with European nationality and residence (94.4 and 97.3%, respectively)—being those associated to Spain the most predominant ones in more than 90% of the sample—with a major representation of the intermediate age range between 31 and 60 years old (58.7%) and with a University educational level (either completed or in progress) (78.4%). All the participants completed all the questionnaires and answered the completeness of the questions.

**Table 1:** Demographics of the study sample.

		**Men**	**Women**	**Total**
Age (years old)	<18	4 (0.5%)	9 (1.1%)	13 (1.6%)
	18–25	32 (4.0%)	96 (11.9%)	128 (15.9%)
	26–30	38 (4.7%)	69 (8.6%)	107 (13.3%)
	31–45	72 (8.9%)	141 (17.5%)	213 (26.4%)
	46–60	88 (10.9%)	173 (21.4%)	261 (32.3%)
	> 60	27 (3.4%)	58 (7.2%)	85 (10.5%)
Educational level	Primary studies	11 (1.4%)	14 (1.7%)	25 (3.1%)
	Secondary studies	16 (2.0%)	14 (1.7%)	30 (3.7%)
	High school	56 (6.9%)	63 (7.8%)	119 (14.8%)
	University student	19 (2.4%)	60 (7.4%)	79 (9.8%)
	University degree	159 (19.7%)	395 (49.0%)	554 (68.7%)
Nationality continent	Europe	250 (31.0%)	512 (63.4%)	762 (94.4%)
	South America	10 (1.2%)	27 (3.4%)	37 (4.6%)
	Central America	1 (0.1%)	5 (0.6%)	6 (0.7%)
	Asia	-	1 (0.1%)	1 (0.1%)
	Africa	-	1 (0.1%)	1 (0.1%)
Residence continent	Europe	254 (31.5%)	531 (65.8%)	785 (97.3%)
	North America	-	1 (0.1%)	1 (0.1%)
	South America	5 (0.6%)	10 (1.2%)	15 (1.9%)
	Central America	2 (0.3%)	3 (0.4%)	5 (0.6%)
	Oceania	-	1 (0.1%)	1 (0.1%)

*Values indicated as absolute and relative frequencies (in parentheses)*.

### Instruments

The Urgency-Premeditation-Perseverance-Sensation seeking (UPPS-P) Impulsivity Scale (Lynam, unpublished; Development of a short form of the UPPS-P Impulsive Behavior Scale. Unpublished technical report) in its reduced and Spanish version (Cándido et al., 2012) is a 20-item questionnaire, which measures 5 traits of impulsivity (4 items each): Negative Urgency, Lack of Premeditation, Lack of Perseverance, Sensation Seeking, and Positive Urgency. Items are answered on a four-point Likert-type scale, from 1 (“strongly agree”) to 4 (“strongly disagree”). The score is inverted on the two scales of Urgency and Sensation seeking so that all of them can be corrected in the direction of impulsivity; each one scoring between 4 and 16. The internal consistency of the 5 scales, which was estimated by using Cronbach's α, ranged from 0.61 to 0.81, with the two Emergency scales below 0.70, which is considered to be the lower admissible limit. In a more recent study in which this questionnaire was applied on the use/abuse of ICT, estimators of internal consistency were applied on the Likert-type scales and acceptable values for all subscales were found (0.75 < ω < 0.89) (Pedrero-Pérez et al., 2020a).

The Obsessive-Compulsive Drug Use Scale (OCDUS) is a 12-item self-report questionnaire. The validation study (Franken et al., 2002) found 3 factors: Thoughts and Interference (6 items), Desire and Control (4 items) and Resistance to Thoughts and Intention (2 items). Responses are given on an analogous 7-points scale (typically ranging from “Not at all” to “All the time”). Items 6 and 12 must be reversed to ensure that all items point toward the same direction. Studies with previous versions showed adequate evidence of internal consistency and validity (Machielsen et al., 2012, 2018; Lievaart et al., 2015). Recently, an OCDUS-ICT version has been proposed to estimate the compulsive use/abuse of ICT (Pedrero-Pérez et al., 2020b), which has shown evidence of reliability (ω < 0.92), and structural and convergent validity.

The screening version of the Prefrontal Symptom Inventory (PSI-20; Pedrero-Pérez et al., 2015), which explores symptoms of malfunction in daily life related to neuropsychological disorders attributable to the prefrontal cortex, is a 20-item scale with a 5-point Likert-type response format (0: “Never or hardly ever”; 1: “A few times”; 2: “Sometimes yes and sometimes no”; 3: “Many times”; 4: “Always or nearly always”). Factorial analysis revealed a three-factor solution: problems in behavioral control, problems in emotional control and problems in social behavior. Adequate internal consistency of all the subscales was reported both in the general population and in addicts under treatment (0.87 < α_s_ < 0.89), as well as in clinical validity tests (Ruiz-Sánchez de León et al., 2015), and cross-cultural validity (González Roscigno et al., 2016; Mendoza et al., 2016; Frontado Frontado, 2019). In the study sample of the present work, the multivariate consistency was α_s_ = 0.1 for the complete test and 0.81 < α_s_ < 0.90 for the scales.

The MULTICAGE-ICT is a 20-item questionnaire consisting of 5 scales designed to investigate problems related to the use of the Internet, cell phone, videogames, instant messaging, and social networks (Pedrero-Pérez et al., 2018). It is based on MULTICAGE CAD-4, a compulsive behavior screening questionnaire, related and non-related to substances (Pedrero-Pérez et al., 2007), which has been used in primary care (Rodríguez-Monje et al., 2009; Reneses et al., 2015; Garrido-Elustondo et al., 2016), behavioral addictions (Estévez Gutiérrez et al., 2014; Estévez et al., 2015; Megías et al., 2018; Jara-Rizzo et al., 2019) and substance addiction (Martínez-González et al., 2013; Navas et al., 2014, 2016). A cell phone use/abuse scale was subsequently included (Rodríguez-Monje et al., 2019). The MULTICAGE-ICT asks four questions with a dichotomous answer (yes/no) for each behavior problem, focusing on: item 1, self-estimated dedication time excess; item 2, estimated dedication time excess by significant others; item 3, difficulty in not performing the behavior; and item 4, difficulties in voluntarily interrupting the behavior. The psychometric study yielded adequate internal consistency of all its scales (0.74 < ω < 0.93) and evidence of structural validity.

### Procedure

Four questionnaires were delivered through Google Docs® platform to obtain the scores for UPPS (20 items), OCDUS (12 items), PSI (20 items), and MULTICAGE-ICT (20 items) questionnaires. The participants for the study were recruited through instant messaging applications (e.g., WhatsApp®, Telegram®), social networks (e.g., Facebook®, Instagram®) and e-mail. At the same time, participants were asked to spread the word to their contacts, using a chain-sampling technique. In the presentation, all participants were informed of the purpose of the study, their explicit consent was requested and were asked to answer as sincerely as possible to all the questions. With no exception, the explicit consent of participating to the study was obtained prior to their enrollment, by directly asking them or to their parents/legal guardians (for underage subjects) to tick the box “I am 14 years old or above and I consent to voluntarily participate in this study.” Participants could only continue with the data submission if they had previously accepted to participate. All the information gathered along the whole study was treated and stored in accordance to the Spanish Digital Data Protection Law of 2018 (“Ley Orgánica 3/2018, de 5 de diciembre, de Protección de Datos y Garantía de Derechos Digitales,” LOPDGDD). Anonymity was guaranteed along the whole process by codifying the answers to prevent their later association to the individuals. Data collection began on the 2nd of January of 2019 and ended on the 3rd of April of 2019. The study was approved by the Formation and Research Unit of Madrid Salud (Ayuntamiento de Madrid).

### Data Analysis

Firstly, the estimation of the minimum sample size was based on the strictest and widely accepted criteria for studies involving questionnaires, which establish an *n*/*p* ratio (*n* = sample size; *p* = number of items included in the analysis) equal to 10 subjects per item (Everitt, 1975). Since 4 questionnaires were employed—three of them with 20 items and one with 12 items—a minimum sample size of 720 subjects was initially estimated. In order to compensate possible later withdrawals (i.e., outliers detection), data collection was maintained until at least *n* = 800 was obtained. The existence of outliers was studied by means of the Mahalanobis distance by applying the *p* < 0.001 criterion for exclusion (De Maesschalck et al., 2000). An *n*/*p* ratio of 11.21 was finally obtained, which was slightly better than that specified by the strictest criteria. No further data cleaning was required, as subject's scores were directly extracted from the delivered questionnaires. A full descriptive analysis was carried out after completely gathering the whole dataset. The quality and authenticity of the data was guaranteed, as well as the reliability of the analyses throughout the entire process. Quantitative variables were synthesized in terms of means, standard deviations, 95% confidence intervals, as well as distribution statistics. Scatter and box plots were employed to represent the detected changes. The normality of the sample distribution was assessed by means of the Kolmogorov-Smirnov test. When appropriate, hypothesis contrast tests (Mann-Whitney *U*-test) were applied in order to demonstrate the existence (or absence) of statistically-significant differences by considering a significance threshold of *p* < 0.05. Depending on the context, the effect size was evaluated through the Rosenthal correlation coefficient (Rosnow et al., 2000) or the Pearson correlation coefficient and interpreted in both cases as: small (*r* < 0.30), medium (0.30 ≤ *r* < 0.50), or large (*r* ≥ 0.50) (Cohen, 1988 and Cohen, 1992). Bayesian factors BF_10_ and 95% credibility intervals were also calculated to support correlation results and interpreted as: no evidence of correlation (BF_10_ < 1), weak to moderate evidence (1 ≤ BF_10_ < 10) or strong evidence (BF_10_ ≥ 10) (Beard et al., 2016). On the other hand, several predictive models were optimized and evaluated (with a previous randomized splitting of the sample into the “training” and “testing” groups, in a respective ratio of 8:2) through supervised Machine Learning algorithms such as simple and multiple linear regression, logistic regression, K-Nearest Neighbor (KNN), Gaussian Process Classification (GPC), Support Vector Machine (SVM), and Decision Tree. All the statistical processing and treatment was carried out in Spyder Anaconda Python 3.7 (Anaconda, Inc.) in conjunction with the Pandas 1.2.2, NumPy v1.20.0, SciPy 1.6.1, Scikit-learn 0.24.1, Pingouin 0.3.10, Matplotlib 3.3.4, and Seaborn 0.11.1 libraries.

## Results

### Determination of the Impulsivity-Compulsivity Axis

According to the definition provided in the Introduction section for the concept of the Impulsivity-Compulsivity axis and the previous methods described by other research groups (Fernández-Serrano et al., 2012), its calculation was uniquely based on the scores of the UPPS and OCDUS contributions, given that they provide with information on the impulsive and compulsive character of the subjects, respectively.

Firstly, all scores were linearly rescaled in a range from 0 (lowest score) to 100 (highest score) (Table 2). Thus, an average close to 36 was obtained for the UPPS contributions and to 10 for the OCDUS contributions.

**Table 2:** Summary of normalized scores for UPPS and OCDUS (*n* = 807).

**Questionnaire**	**Mean**	**Standard deviation**	**Median**	**Minimum–Maximum**
**UPPS**				
Negative urgency	47.41	18.49	41.67	25.00–100.00
Positive urgency	45.97	15.27	41.67	16.67–100.00
Lack of premeditation	24.47	16.39	25.00	0.00–75.00
Lack of perseverance	20.62	16.83	16.67	0.00–75.00
Sensation seeking	43.33	19.60	41.67	16.67–100.00
**OCDUS**				
Thought interference	14.06	5.65	13.00	6.00–37.00
Desire control	11.46	4.75	11.00	4.00–28.00
Resistance	4.07	2.29	3.00	2.00–14.00

In order to detect possible interferences caused by the demographic variations of the sample, the existence of correlations between age and educational level with respect to UPPS and OCDUS scores was verified (Table 3). Thus, it was observed that both tests were significantly correlated with age, while only UPPS (with the exception of Lack of premeditation) was correlated with the educational level. Nevertheless, it should be noted that such correlations were weak in all cases (Pearson's coefficients ranging from−0.35 ≤ r ≤ 0.19, Table 3), which suggested a low effect size. Despite this fact, we found it appropriate to transform the results into standardized scores. To do so, the corresponding linear regression lines between age and educational level against UPPS and OCDUS scores were optimized. Later on, the standardized residuals were calculated and, finally, the corresponding Z scores were extracted from the means and associated standard deviations.

**Table 3:** Correlations between UPPS and OCDUS scores vs. demographic variables of age and educational level, expressed in terms of *p*-value, Pearson *r* coefficient, Bayesian BF_10_ coefficient and 95% credibility intervals (CI).

**Questionnaire**	**Age**				**Educational level**			
	***p* value**	**Pearson *r***	**BF_**10**_**	**CI 95%**	***p* value**	**Pearson *r***	**BF_**10**_**	**CI 95%**
**UPPS**								
Negative urgency	0.04	−0.07	0.34	−0.14,−0.00	<0.001	−0.16	1303	−0.23,−0.09
Positive urgency	<0.001	−0.19	9 × 10^4^	−0.25,−0.12	<0.001	−0.17	7364	−0.24,−0.1
Lack of premeditation	0.01	−0.09	0.92	−0.15,−0.02	0.25	0.04	0.09	−0.03, 0.11
Lack of perseverance	0.01	−0.09	1.54	−0.16,−0.03	<0.01	−0.11	5.13	−0.18,−0.04
Sensation seeking	<0.001	−0.35	7 × 10^20^	−0.41,−0.28	0.02	−0.09	0.87	−0.18,−0.04
**OCDUS**								
Thought interference	<0.001	−0.28	9 × 10^12^	−0.34,−0.22	0.26	−0.04	0.08	−0.11, 0.03
Desire control	<0.001	−0.28	5 × 10^12^	−0.34,−0.21	0.83	0.01	0.04	−0.08, 0.06
Resistance	<0.001	−0.35	1 × 10^21^	−0.41,−0.29	0.76	−0.01	0.05	−0.05, 0.09

After standardizing the scores of all UPPS and OCDUS contributions, we proceeded to calculate a new indicator that could serve as a good representative of the impulsive and compulsive nature of each individual. More concretely, such indicator was obtained by, firstly, calculating the arithmetic mean of the 5 UPPS contributions (Negative Urgency, Positive Urgency, Lack of Premeditation, Lack of Perseverance and Sensation Seeking) for the impulsive character and, on the other hand, the arithmetic mean of the 3 OCDUS contributions (Thought Interference, Desire Control and Resistance) for the compulsive character. Secondary, the location of each subject on the Impulsivity-Compulsivity axis was determined by direct subtraction of the calculated compulsive character from the impulsive one. Likewise, in order to obtain a better resolution of the variable and more easily appreciate the differences between subjects, the result was multiplied by 10.

**Figure 1:**
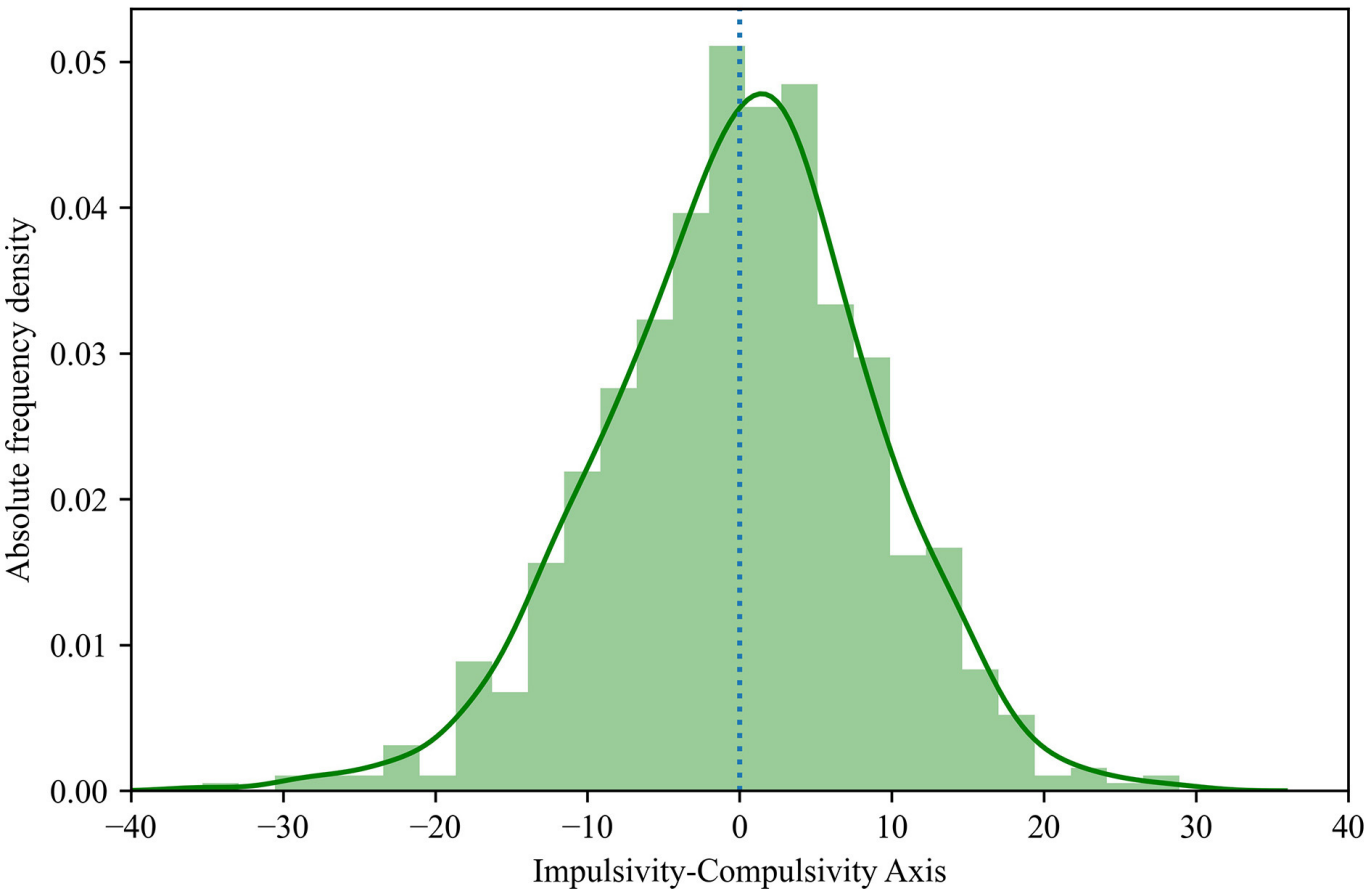
Population histogram of the calculated Impulsivity-Compulsivity axis.

Among the results obtained, the most positive values of the Impulsivity-Compulsivity axis were associated to impulsive characters and, conversely, the most negative ones to individuals more prone to compulsivity. In this sense, qualitative ranges were established to facilitate the analysis of the sample: (1) values above +20 were associated to “high impulsivity,” (2) values between +10 and +20 (inclusive) to “moderate impulsivity,” (3) values between−10 and +10 (both inclusive) to “character balance,” (4) values between−10 and−20 (inclusive) to “moderate compulsivity,” and (5) values below−20 to “high compulsivity.” At this point and for the obtained sample, an overall average of 0.00 was detected in relation to the calculated Impulsivity-Compulsivity axis with a standard deviation of 8.84 (Table 4). It could be observed that the values of the axis were symmetrically-distributed around the mean (Figure 1), with a global balance of impulsivity and compulsivity (none of the two characters was predominant), as confirmed by the low asymmetry coefficient (-0.23).

**Table 4:** Statistical summary of the values found for the Impulsivity-Compulsivity axis.

**Statistic**	**Impulsivity-Compulsivity axis**
*N*	807
Mean	0.00
Standard deviation	8.84
Minimum	−35.30
Quartile 1	−5.65
Quartile 2 (Median)	0.30
Quartile 3	5.80
Maximum	28.90
Asymmetry coefficient	−0.23
Kurtosis coefficient	0.51

**Figure 2:**
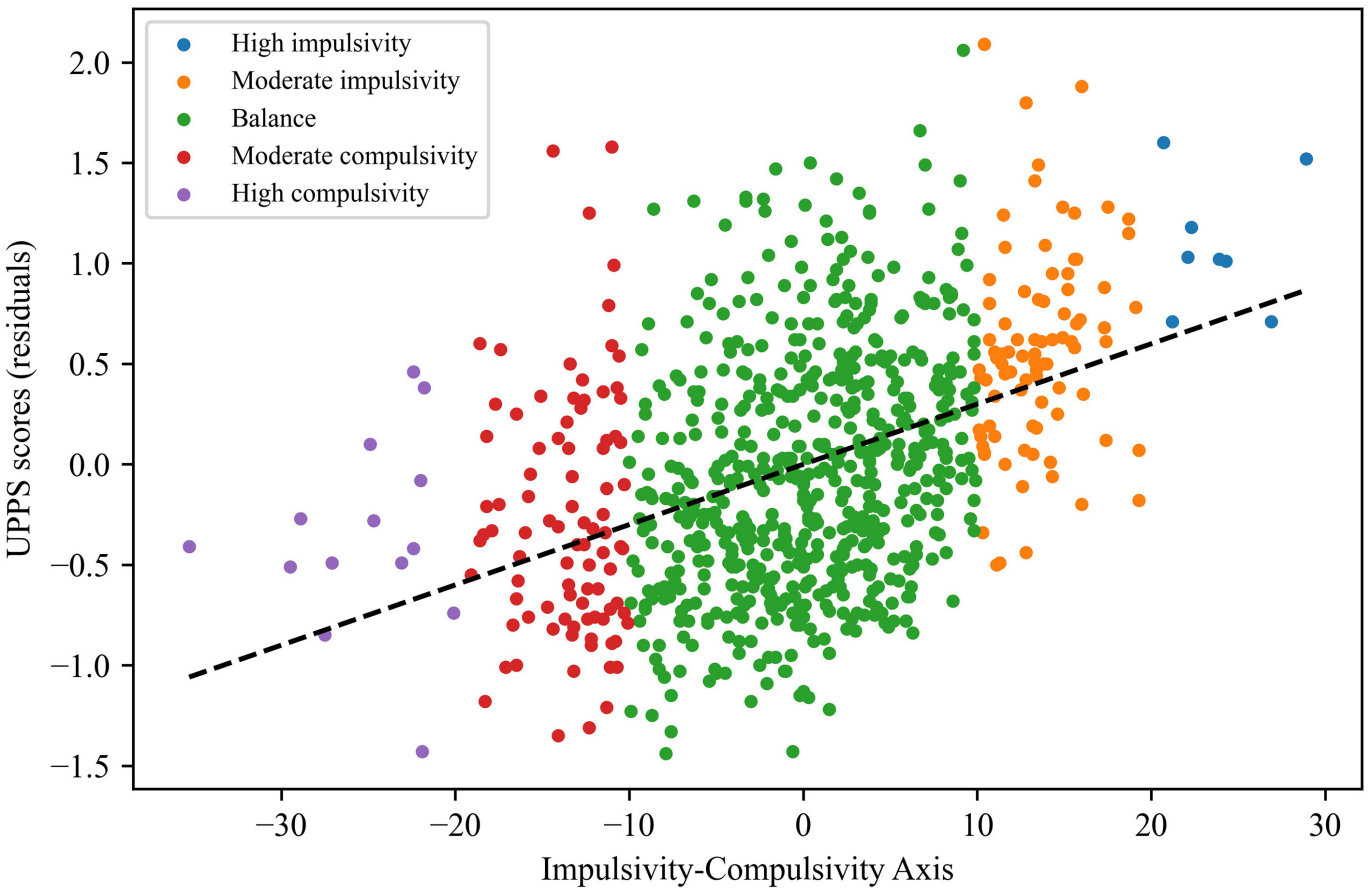
Scatter plot of UPPS scores (residuals) vs. the calculated values of the Impulsivity-Compulsivity axis.

**Figure 3:**
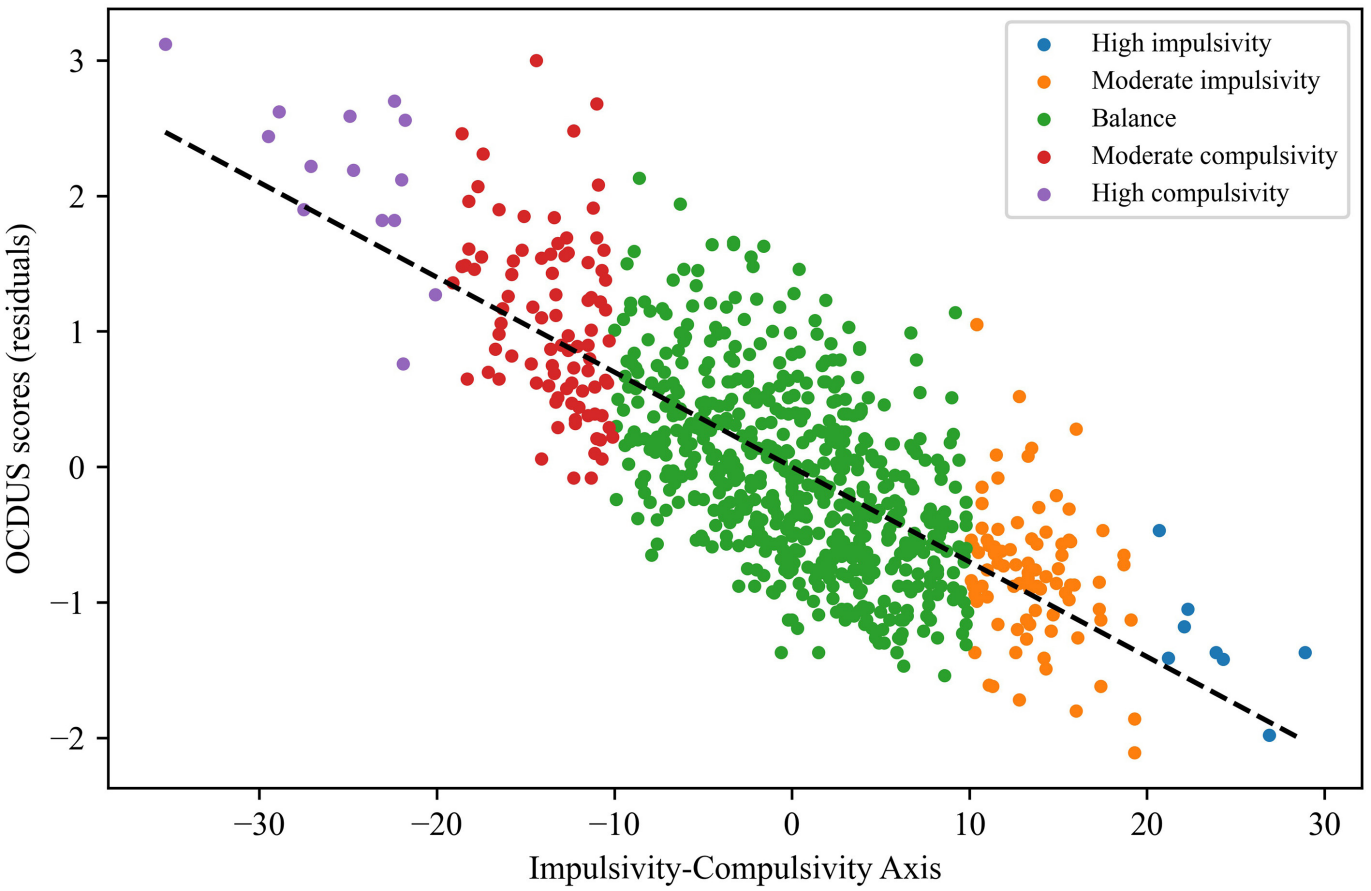
Scatter plot of the OCDUS scores (residuals) vs. the calculated values of the Impuslsivity-Compulsivity axis.

**Figure 4:**
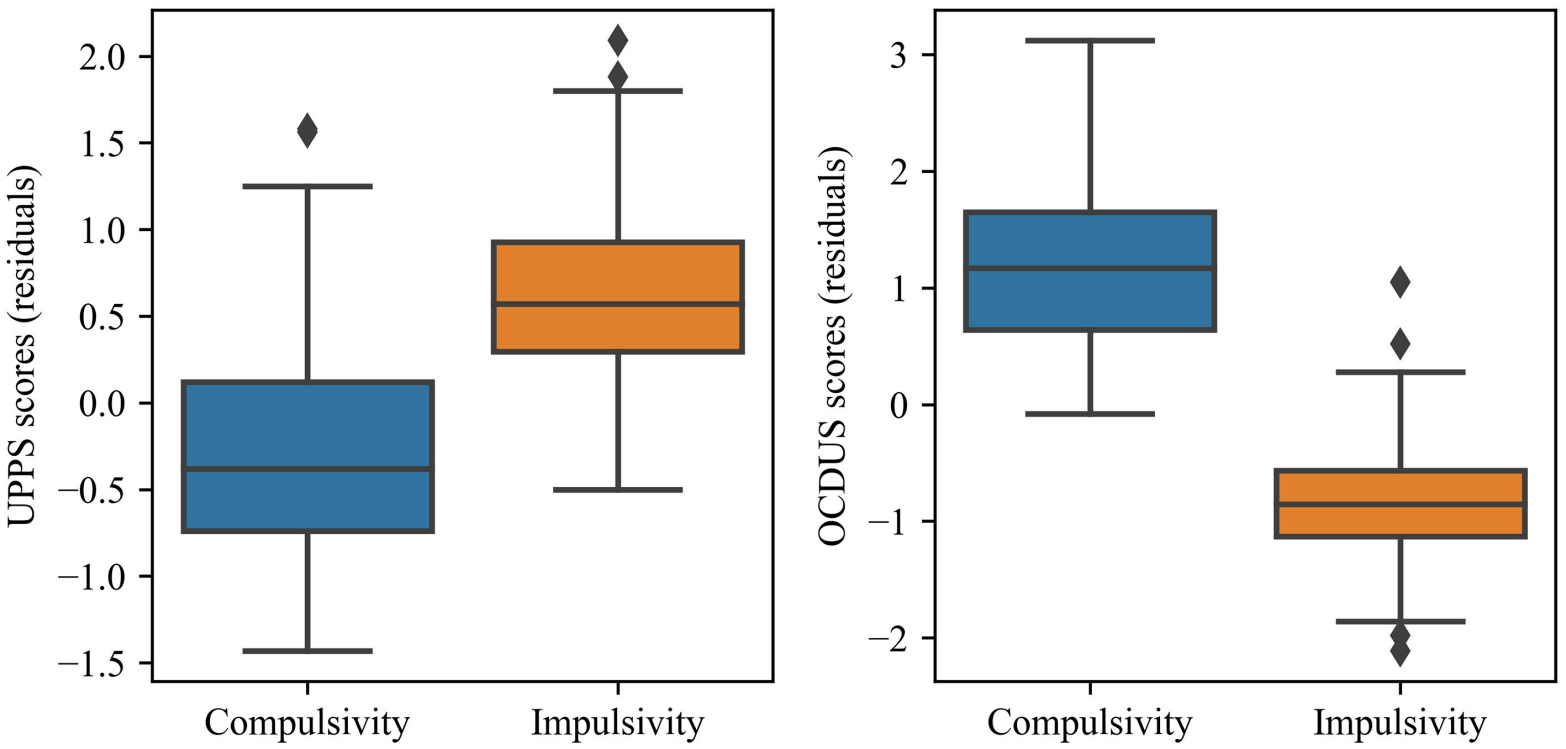
Box-plot of UPPS and OCDUS scores (residuals) according to the type of character of the subjects.

On the other hand, the internal consistency of the axis was verified through the comparison of the calculated values with respect to the residuals obtained for the UPPS and OCDUS scores (Figures 2, 3). More specifically, we found that the highest axis values were associated to the highest UPPS scores (upward trend) and, at the same time, to the lowest OCDUS scores (downward trend). The existence of such trends could be demonstrated through the corresponding correlation coefficients for UPPS (Pearson's coefficient = 0.42; *p* < 0.001) and OCDUS (Pearson's coefficient = −0.73; *p* < 0.001) scores. Moreover, after checking that the evaluated datasets were non-normally distributed according to the Kolmogorov-Smirnov test, the application of the Mann-Whitney's *U*-test (α = 0.05), allowed us to conclude that statistically-significant differences (*p* < 0.001) existed between the UPPS and OCDUS scores for impulsive vs. compulsive subjects, with large effect sizes (*r* > 0.60) in both cases according to Rosenthal correlation coefficient (Figure 4, Table 5).

**Table 5:** Analysis of statistically-significant differences between the UPPS and OCDUS scores for impulsive and compulsive subjects.

		**Questionnaire**	
		**UPPS**	**OCDUS**
Impulsive subjects	Mean	0.618	−0.814
	Median	0.570	−0.855
	Standard deviation	0.531	0.550
	Interquartile range	0.632	0.565
Compulsive subjects	Mean (%)	−0.280	1.220
	Median (%)	−0.380	1.170
	Standard deviation	0.594	0.741
	Interquartile range	0.860	1.010
Mann-Whitney *U* test	*U* statistic	1256.0	137.0
	*p* value	<0.001	<0.001
Rosenthal correlation	*r* coefficient	0.630	0.840

*Descriptive statistics expressed as score residuals*.

In this context, we decided to analyze in more detail the subpopulations composed by individuals with addiction signs in order to know whether their character was generally more prone to impulsivity or to compulsivity. To carry out such analysis, the scores of the MULTICAGE-ICT test were taken into account to establish the subpopulations by considering that an individual might present addiction signs if the test afforded a value equal to or greater than 50%. The results of this evaluation (Table 6) suggested that individuals with more probabilities of being addicts according to the MULTICAGE-ICT test were statistically more prone to compulsivity (more specifically, to a “moderate” compulsivity) than to impulsivity. Likewise, it could be demonstrated through the Mann-Whitney's *U* test (α = 0.05) that statistically-significant differences (*U* = 25.0, *p* = 0.032) existed between the proportions of both characters, with a medium effect size as stated by the Rosenthal correlation coefficient (*r* = 0.410). Therefore, the number of addicted individuals with compulsive behavior (mean = 10.860%, median = 8.645%, standard dev. = 8.236%, interquartile range = 14.605%) was objectively higher than that of individuals with impulsive character (mean = 4.873%, median = 4.145%, standard dev. = 4.312%, interquartile range = 6.910%).

**Table 6:** Percentage frequency (%) of each band of the Impulsivity-Compulsivity axis according to the subpopulations of the MULTICAGE-ICT test.

**Impulsivity-Compulsivity axis**	**MULTICAGE-ICT test**				
	**Internet**	**Cell phone**	**Videogames**	**Instant messaging**	**Social networks**
High impulsivity	1.22	0.22	1.30	1.02	1.08
Moderate impulsivity	6.99	8.19	11.69	9.49	7.53
Balance	68.39	72.12	71.43	66.78	63.98
Moderate compulsivity	20.06	16.59	12.99	18.64	23.12
High compulsivity	3.34	2.88	2.60	4.07	4.30

### Impulsive-Compulsive Character Prediction

Based on the features of the Impulsivity-Compulsivity axis found with the current study sample, we were encouraged to elucidate an heuristic model capable of predicting whether a patient can be more likely classified as impulsive or compulsive.

To achieve such goal, the PSI and MULTICAGE-ICT scores provided by the subjects were used to optimize various possible models. Moreover, those subjects that had been classified as “balance character” according to the calculated Impulsivity-Compulsivity axis (values between−10 and +10) were excluded for this procedure in order to maximize the efficiency of the models optimization. In this way, the sample was reduced to 201 subjects (24.9% of the initial number 807). Finally, and with the aim of optimally evaluating the real efficacy of the models found, the study sample (*n* = 201) was randomly divided into a training set (*n* = 161) and a test set (*n* = 40).

After fitting and optimizing the data into the most common supervised Machine Learning models (single and multiple linear regression, logistic regression, KNN, GPC, SVM, and Decision Tree), we concluded that the GPC model was the most suitable and best adjusted one to fit the distribution of the study sample. The optimal parameters for the GPC model entailed: RBF (1.0) kernel, L-BFGS-B optimizer, 100 maximum iterations and without warm start.

**Figure 5:**
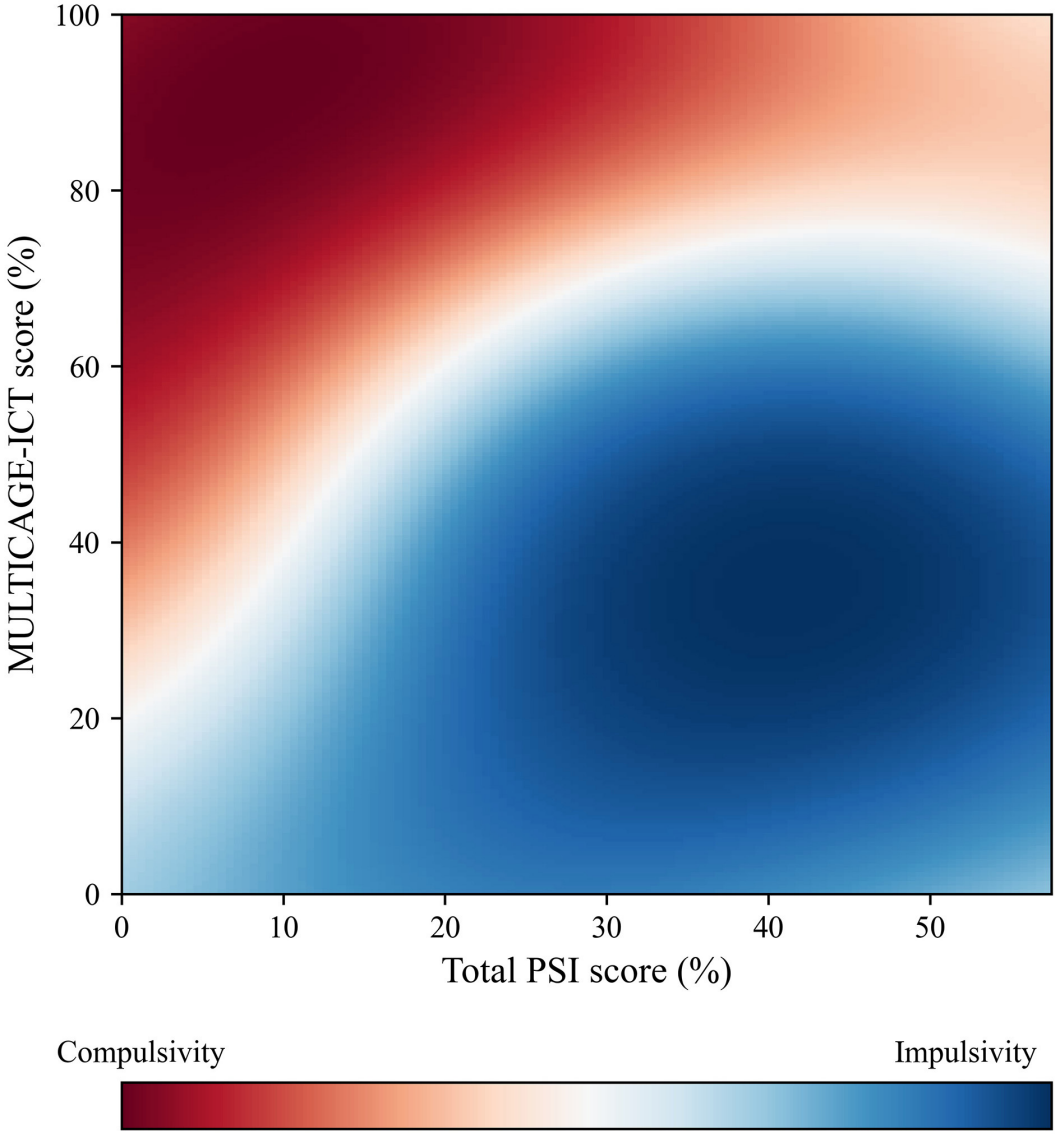
GPC_PSI-MC scatter plot to predict if addicted subjects display an impulsive or compulsive character based on PSI and MULTICAGE-ICT (78% predictive efficiency).

More specifically, a prediction efficiency of 78.1% to predict the impulsive/compulsive character of the subjects was achieved by combining the total PSI and the MULTICAGE-ICT scores (vs. the prediction efficiencies lower than 70% for the rest of the supervised models). Using the scatter plot prepared from the optimized GPC model (Figure 5, from now on referenced as GPC_PSI-MC) it became possible to predict whether a potentially-addicted individual (either to social networks, instant messaging, videogames, Internet, or cell phones) was most likely impulsive or compulsive based on his MULTICAGE-ICT and total PSI scores. In this context, up to 3 probable scenarios would emerge: (1) if a subject obtains a relatively high PSI score and a relatively low score in the MULTICAGE-ICT test, it can be seen that such combination would fall into the blue zone (Figure 5), so that the individual would be probably more prone to an impulsive character; (2) if an individual scores relatively low in total PSI and high in MULTICAGE-ICT, he would be probably defined as a compulsive subject; or alternatively (3) it could be assumed that the PSI—MULTICAGE-ICT combination could fall in a white-colored zone (Figure 5), entailing that the classification of the subject's character would be uncertain.

**Figure 6:**
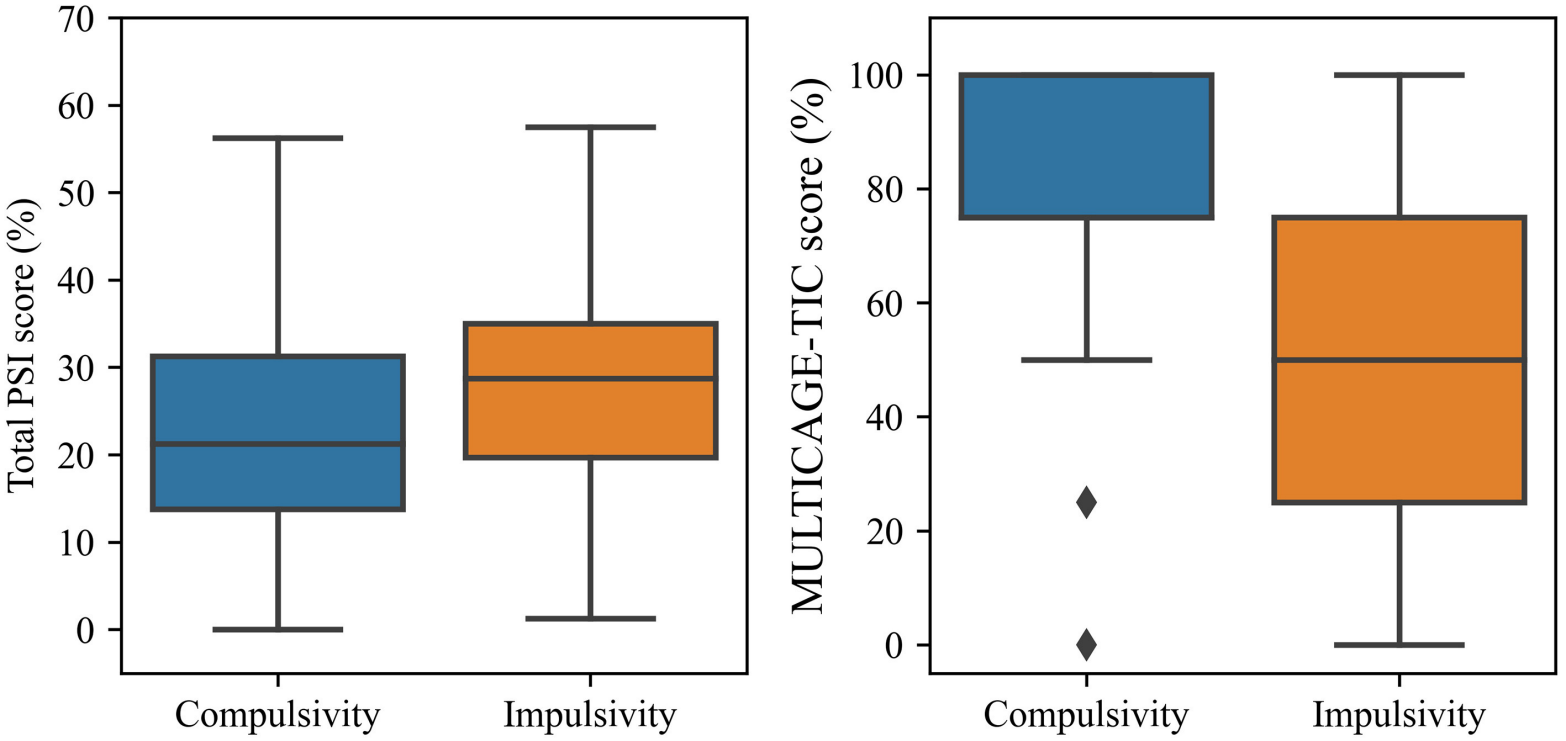
Box-plot of PSI-20 and MULTICAGE-ICT scores according to the type of character of the subjects.

Based on GPC_PSI-MC, it could be extracted that individuals that score high in the MULTICAGE-ICT test tended to more likely correspond to compulsive characters, while those that score low were associated to impulsive characters (Figure 6, Table 7). Such fact was corroborated by the statistically-significant differences (*U* = 3071.0, *p* < 0.001) detected in this regard through the Mann-Whitney's *U* test (α = 0.05) (after checking that the datasets were non-normally distributed with the Kolmogorov-Smirnov test) and the large effect size found by means of the Rosenthal correlation coefficient (*r* = 0.520). On the other hand, higher total PSI scores may be associated to both compulsive and impulsive characters, though there was a slightly higher predominance toward impulsivity; while low total PSI scores predominantly lead to compulsive characters (Figure 6, Table 7). Furthermore, statistically-significant differences in this regard through the Mann-Whitney's *U*-test (α = 0.05) were found (*U* = 3572.5, *p* < 0.001) and the Rosenthal correlation coefficient revealed the presence of a small effect size (*r* = 0.250).

**Table 7:** Analysis of statistically-significant differences between the PSI and MULTICAGE-ICT scores for impulsive and compulsive subjects.

		**Questionnaire**	
		**PSI**	**MULTICAGE-ICT**
Impulsive subjects	Mean	28.294	47.396
	Median	28.750	50.000
	Standard deviation	11.673	28.527
	Interquartile range	15.312	50.000
Compulsive subjects	Mean (%)	22.440	75.952
	Median (%)	21.250	75.000
	Standard deviation	12.441	22.448
	Interquartile range	17.500	25.000
Mann-Whitney *U* test	*U* statistic	3572.5	3071.0
	*p* value	<0.001	<0.001
Rosenthal correlation	*r* coefficient	0.250	0.520

*Descriptive statistics expressed as score percentages (%)*.

**Figure 7:**
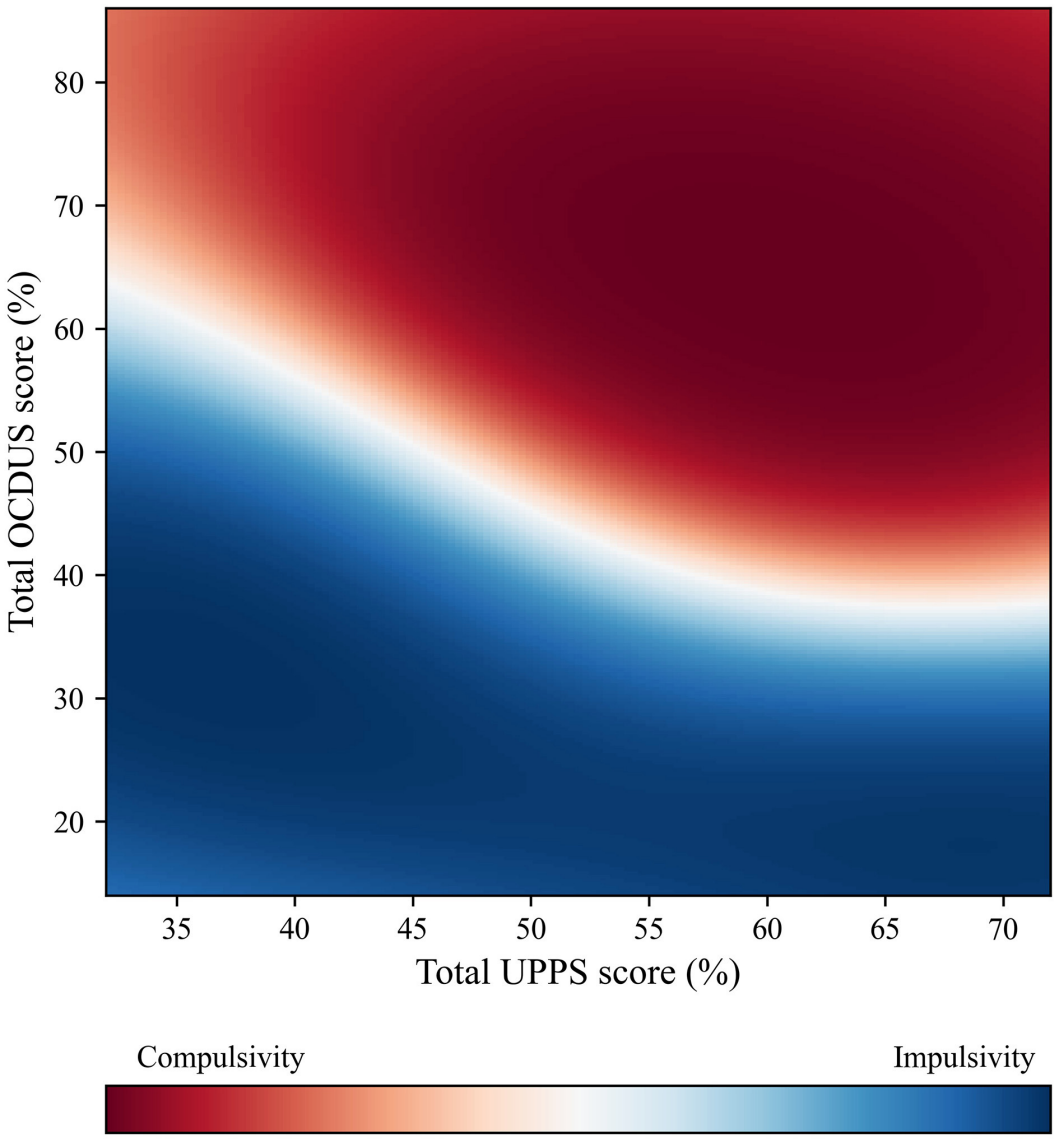
GPC_UPPS-OCDUS scatter plot to predict if addicted subjects display an impulsive or compulsive character based on UPPS and OCDUS scores (88% predictive efficiency).

Analogously, the use of the same analytical methodology made it possible to optimize a second GPC model based on the UPPS and OCDUS scores. The study sample consisted of the 201 subjects as well, which had not been classified as “balance character” according to the calculated axis, and were randomly divided into a training set (*n* = 161) and a test set (*n* = 40). The optimized result achieved for this second GPC model (from now on referenced as GPC_UPPS-OCDUS) could be represented through the scatter plot presented in Figure 7, which exhibits a predictive efficiency of 87.8%.

**Figure 8:**
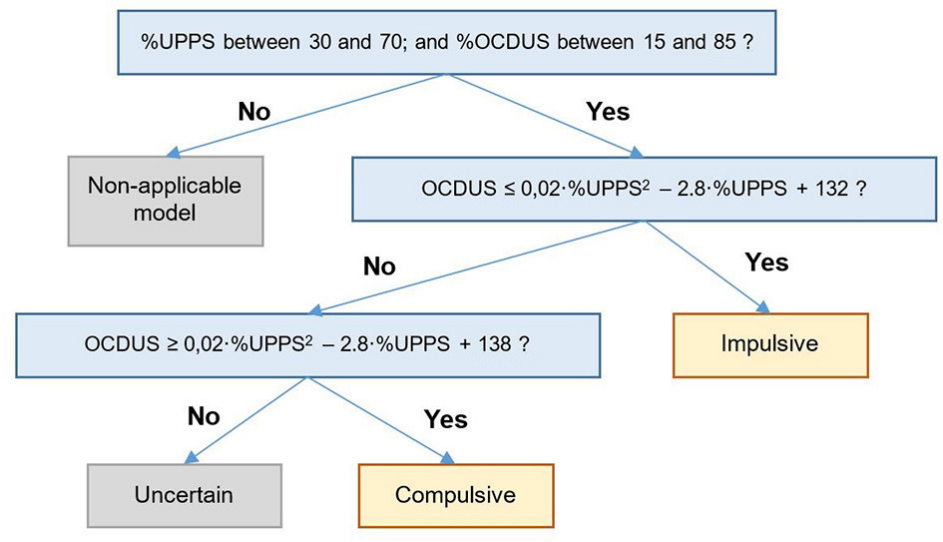
Decision tree based on the GPC_UPPS-OCDUS model to predict whether an addicted subject has an impulsive or compulsive character based on UPPS and OCDUS (80% predictive efficiency).

The GPC_UPPS-OCDUS model made it possible to predict whether a potentially-addicted individual was more prone to an impulsive or compulsive character based on the total scores of the UPPS and OCDUS tests. According to this model, up to 3 possible scenarios might occur: (1) if a subject scores relatively low in OCDUS (<30%), he/she would probably exhibit an impulsive character when, as he/she would fall in the blue zone (Figure 7); (2) if a subject scores relatively high in OCDUS (more than 70%), his/her character would predictably be compulsive, since he/she would be located in the red zone (Figure 7); and (3) if the subject exhibits intermediate OCDUS scores (between 30 and 70%), the prediction of his/her character would be strongly determined by the UPPS value. Owing to the excellent efficiency achieved by GPC_UPPS-OCDUS, we were encouraged to simplify the previous model by preparing a simple decision tree (Figure 8), which makes it possible to locate a subject that has completed the UPPS and OCDUS test on the calculated Impulsivity-Compulsivity axis. This decision tree presents a reliability of 80.1% and entails the comparison of the corresponding OCDUS scores with the result of two formulas, which are representative of the quadratic character that follows the borderline between the impulsive and compulsive characters on the scatter plot of the GPC_UPPS-OCDUS model (Figure 7).

## Discussion

In this work, the use of UPPS, OCDUS, PSI and MULTICAGE-ICT questionnaires provided useful information to assess the impulsive/compulsive behavior of 807 subjects who suffer from ICT addiction. Thanks to the collected data, their addiction features could be successfully analyzed and two mathematical models were devised to facilitate a rapid diagnose of impulsivity or compulsivity based on the PSI and MULTICAGE scores or, alternatively, the UPPS and OCDUS scores.

The results obtained in the present study provide reasonable evidence of the existence of an Impulsivity-Compulsivity axis in the process of addiction to ICT. This evolutionary axis was proposed in the neuropsychological context of addiction formulated by Everitt and Robbins (2005, 2013), according to which the controlled use initially turns into an impulsive use and later becomes a compulsive use; all related to changes in the cerebral location of the control mechanisms. If these indications were confirmed, it would be possible to classify a subject seeking treatment from the scores obtained in the four self-questionnaires used in this study, which would allow therapeutic methods to be adapted to their specific evolutionary moment.

To carry out this study, we employed two questionnaires proposed by a large international consensus group in the context of the RDoC (Insel et al., 2010), which is responsible of identifying the central neuropsychological constructs of addiction and the most appropriate instruments to measure them (Yücel et al., 2019). UPPS was proposed as a suitable instrument to measure response inhibition and OCDUS to measure compulsivity. Moreover, an additional questionnaire was employed to measure malfunctioning symptoms of the prefrontal cortex (PSI), which is involved in both processes, and a questionnaire on the use/abuse of ICTs, which explores and classifies the behavior of the subjects in the use of these virtual environments and their applications.

Since nearly no previous attempts to estimate the Impulsivity-Compulsivity evolutionary axis exist to date, a procedure has been designed taking as a base the initially-proposed one for the first of these studies (Fernández-Serrano et al., 2012). Thus, it has been possible to define and establish a calculation method to obtain a representative variable of the Impulsivity-Compulsivity axis through the standardization of UPPS and OCDUS scores and their subtraction, as well as the application of Machine Learning techniques. The calculated axis was consistent with the initial test scores, as it confirms that the more impulsive an individual is, the higher his UPPS score and the lower his OCDUS score. Conversely, the more compulsive a character, the higher the OCDUS scores and the lower the UPPS scores. Additionally, it has been possible to optimize two models capable of predicting whether a potentially-addicted individual is likely to exhibit a character closer to compulsivity or impulsivity with efficiencies >80%.

According to this analytical model and stated in a generic way, the impulsive or compulsive status of an individual cannot be solely predicted through the UPPS or OCDUS taken separately, but it is necessary to analyze the combination of these scores, except for the extreme OCDUS scores. Similarly, an individual's impulsive or compulsive condition cannot be predicted simply through the PSI value, which only reports symptoms of prefrontal malfunctioning without pointing toward one or the other extreme, nor from the MULTICAGE-ICT, but it is necessary as well to analyze which combination arises from these two scores. High PSI scores tend to be associated to impulsive behaviors, while low scores tend to point toward compulsive behaviors. The interpretation of the scatter plots prepared for this model leads to the fact that individuals with lower PSI scores tend to become compulsive earlier for less intense addictions (low MULTICAGE-ICT score). Conversely, if an individual displays a high PSI value, his transition from impulsivity to compulsivity only occurs when his addiction is much stronger (high MULTICAGE-ICT score). As suggested by the theoretical model, it can be rationalized that an addiction always begins being mild and, afterwards, it gradually increases its intensity (passing from 0 to 1 scores in the MULTICAGE-ICT test to higher values): the model justifies that the individual initially behaves impulsively (low MULTICAGE-ICT) and later transitions toward compulsivity when his addiction becomes stronger (high MULTICAGE-ICT).

On the other hand, the data from the present study suggest that higher ICT abuse scores point more probably toward compulsivity. OCDUS scores are more decisive than the UPPS ones, since a value higher than 70% of OCDUS is already indicative of compulsivity, while a value lower than 30% is indicative of impulsivity. These findings, which imply a more compulsive than impulsive character of ICT abuse, replicate previous studies carried out on a similar sample and different methods of analysis (Pedrero-Pérez et al., 2020a,b) and oppose those encountered in previous studies that overestimate the role of impulsivity in front of the less-explored compulsivity (Lee et al., 2019).

The study of behavioral addictions has given more importance to compulsivity than to substance addiction, probably due to the temporal coincidence with the growing interest of this concept. Thereby, compulsivity is considered one of the central constructs of the addiction to smartphones (Lin et al., 2017) to the point that, when the “addiction” term is avoided, the “compulsive use” term is preferred (i.e., Thomas and Hajiyev, 2020; Wang and Lee, 2020). Nevertheless, the predominance of the “compulsivity” concept linked to the use/abuse of ICT does not seem to be justified by scientific findings, but by a mere terminological preference in the research of names that elude the disputed addictive character of such behaviors. It is common to find ambiguous concepts in the literature, such as “problematic use” (Meng et al., 2020; Busch and McCarthy, 2021). Consequently, more studies are required to explore the neuropsychological perspectives of the compulsivity construct to clarify the terminological chaos that affects this study context.

The main limitations of the presented study are related to the recruitment method: The use of online surveys presents significant risks that should be minimized as much as possible. However, once these risks are known, this type of sampling has proliferated in recent years, allowing large samples to be quickly and efficiently obtained (Evans and Mathur, 2018). In this study, the consistency of the responses was controlled by means of an outliers detection procedure, obtaining that more than 99% of the responses were consistent. A remarkable issue encountered in this study was the sample size reduction when optimizing the GPC_PSI-MC and GPC_UPPS-OCDUS models (from the initial 807 subjects sample to 201), as only those patients that exhibited a non-equilibrium Impulsivity-Compulsivity axis value were taken into account. In this sense, even though most subjects were excluded for this procedure, the still remaining 201 were considered enough to prepare preliminary models and to assess the feasibility of predicting the impulsive/compulsive character. Of course, the results cannot be generalized in terms of prevalence of each question, but they do allow the study of the relationships between variables, as it was the purpose of this study. Future studies should be aimed at looking for sampling methods capable of obtaining generalizable results.

In conclusion, the data of the present study allowed us to find reasonable indications of the existence of an Impulsivity-Compulsivity axis in the use/abuse addiction to ICTs, consistent with the neuropsychological proposal of Everitt and Robbins. Despite being a preliminary study, the identification of this axis and the location of each subject along this continuum would allow clinicians to assign the most appropriate psychological and/or pharmacological therapeutic intervention (matching), which would undoubtedly improve the results of the treatment.

Given that the contribution of this study was carried out with a non-randomized sample of ICT users, it would be necessary not only to improve the sampling procedures, but also to extend it to samples of subjects with substance use/abuse addiction, as well as to explore whether the use of other types of tests (neuropsychological, neuroimaging, etc.) would improve the predictive capacity of the model and improve the assignment of subjects that are in this undefined zone. Finally, addiction could be regarded as a dimensional evolutionary process and not as a dichotomous category, as it is currently considered in diagnostic classifications.

In order to facilitate the use of the GPC_PSI-MC and GPC_UPPS-OCDUS models in the clinical practice, an IT application has been released (available from DOI 10.6084/m9.figshare.14073980). Thanks to this tool, the introduction of the PSI/MULTICAGE-ICT or UPPS/OCDUS scores allows a rapid prediction if a potentially-addicted patient is closer to an impulsive or a compulsive character, as well as with what probability.

The full database and analytic scripts can be downloaded from *DOI* 10.6084/m9.figshare.13663667 and *DOI* *10.6084/m9.figshare.14073935, respectively*.

## Data Availability Statement

The raw data supporting the conclusions of this article was made available by the authors, without undue reservation.

## Ethics Statement

Ethical approval was not provided for this study on human participants because the anonymous and voluntary collaboration of the subjects was requested. No intervention was made on the participants. Written informed consent for participation was not provided by the participants' legal guardians/next of kin because this was included in the application used for data collection and only if the participant accepted could continue with the data submission.

## Author Contributions

DC-P performed statistical analysis and applied Machine Learning procedures. EP-P performed the data collection procedure and the theoretical formulation of the research. All authors have collaborated in the design of the research and the elaboration of the article.

## Conflict of Interest

DC-P was employed by Banco Santander. The remaining authors declare that the research was conducted in the absence of any commercial or financial relationships that could be construed as a potential conflict of interest.
